# Conference Calendar

**DOI:** 10.1002/cfg.203

**Published:** 2002-10


					Comparative and Functional Genomics
Comp Funct Genom 2002; 3: 459-460.
Published online 29 August 2002 in Wiley InterScience (www.interscience.wiley.com). DOI: 10.1002/cfg.203
Conference Calendar
January-March 2003
Bacteria
SFAM -- Lab on a Chip -- Jan 8-9
http://www.sfam.org.uk/
Bioinformatics
Pacific Symposium on Biocomputing (PSB
2003) -- Jan 3-7
http://psb.stanford.edu/
Keystone -- Computational Biology of Time
-- Jan 31-Feb 4
http://www.symposia.com/Meetings/
ViewMeetings.cfm?MeetingID = 659
Functional Genomics
Keystone -- Gene Suppression: Drug Target
Validation -- Jan 17-22
http://www.symposia.com/Meetings/
ViewMeetings.cfm?MeetingID = 624
Keystone -- Functional Genomics: Global
Analysis of Complex Biological Systems -- Feb
20-25
http://www.symposia.com/Meetings/
ViewMeetings.cfm?MeetingID = 647
ABRF2003: Translating Biology Using
Proteomics and Functional Genomics -- March
1-4
http://www.faseb.org/meetings/abrf2003/
General Genomics
Keystone -- Transposition and Other Genome
Rearrangements -- Feb 8-14
http://www.symposia.com/Meetings/
ViewMeetings.cfm?MeetingID = 636
GRC -- Quantitative Genetics And Genomics
-- Feb 9-14
http://www.grc.uri.edu/03sched.htm
CSHL -- The Biology of DNA -- Feb
26 -- March 2
http://meetings.cshl.org/2003helix.htm
Multiorganism
Plant and Animal Genomes XI Conference
-- Jan 11-15
http://www.intl-pag.org/pag/
Pharmacogenomics
50 Years On: From The Double Helix To
Molecular Medicine -- Feb 1-5
http://www.med.miami.edu/mnbws/
IBC -- InfoTechPharma -- Feb 10-13
http://www.infotechpharma.com/
CHI -- Molecular Medicine Marketplace (was
Tri-Genome) -- March 17-21
http://www.chimolecularmed.com/
IBC -- ScreenTech -- March 24-27
http://www.lifesciencesinfo.com/screentech/
Plants
22nd Symp. Plant Biology: Frontiers of Plant
Cell Biology -- Jan 15-18
http://www.cepceb.ucr.edu/news/news.htm#1
Copyright  2002 John Wiley & Sons, Ltd.
460 Conference Calendar
GRC -- Temperature Stress In Plants -- Jan
26-31
http://www.grc.uri.edu/03sched.htm
Monocots III -- March 31-April 5
http://www.monocots3.org/
Proteomics
CHI -- The Protein Information Week
(PepTalk) -- Jan 13-16
http://www.chi-peptalk.com/
Keystone -- Membrane Proteins: Structure
and Mechanism -- Feb 4-10
http://www.symposia.com/Meetings/
ViewMeetings.cfm?MeetingID = 632
Keystone -- Proteomics: Technologies and
Applications -- March 25-30
http://www.symposia.com/Meetings/
ViewMeetings.cfm?MeetingID = 654
ICES -- Proteomics: Present Perspectives,
Future Challenges -- Mar 28-30
http://www.proteinworks.com/bes/
conferences.html
5th European Symposium of the Protein
Society -- March 29-April 2
http://www.faseb.org/meetings/euro ps/
Structural Genomics
Keystone -- Frontiers of NMR in Molecular
Biology VIII -- Feb 4-10
http://www.symposia.com/Meetings/
ViewMeetings.cfm?MeetingID = 633
Transcriptomics
GRC -- RNA Editing -- Jan 19-24
http://www.grc.uri.edu/03sched.htm
CHI -- Informatics And Microarray Data
Analysis -- Feb 11-12
http://www.healthtech.com/2003/mde/index.htm
CHI -- 5th Lab-On-A-Chip And Microarrays
Europe -- Feb 13-14
http://www.healthtech.com/2003/mfe/index.htm
Key
CHI -- Cambridge Healthtech Institute: http://
www.healthtech.com/
CSHL -- Cold Spring Harbour Laboratory: htpp://
www.cshl.org/
GRC -- Gordon Research Conferences: http://
www.grc.uri.edu/
IBC -- IBC Conferences: http://www.ibcusa.com/
ICES -- International Council of Electrophoresis
Societies: http://www.aesociety.org/ICES.html
Keystone -- Keystone Symposia: http://www.
symposia.com/
SFAM -- Society for Applied Microbiology:
http://www.sfam.org.uk/
Copyright  2002 John Wiley & Sons, Ltd. Comp Funct Genom 2002; 3: 459-460.

				

